# Supplementary material for: A generic battery-cycling optimization framework with learned sampling and early stopping strategies
Source: Patterns (N Y). 2022 Jun 20;3(7):100531. doi: 10.1016/j.patter.2022.100531 (PMC9278511; doi:10.1016/j.patter.2022.100531)
Supplement: Document S1. Figure S1 and Table S1 [file mmc1.pdf]

Patterns, Volume *3*

Supplemental information

A generic battery-cycling optimization framework with learned sampling and early stopping strategies

Changyu Deng, Andrew Kim, and Wei Lu

Supplemental Experimental Procedures

Contents

1. Experimental
  1.1. Battery cells
  1.2. Calendar aging
  1.3. Check-up procedure
  1.4. Data processing
2. Degradation model
  2.1. Pseudo two-dimensional (P2D) model
  2.2. Degradation mechanisms
  2.3. Implementation details

1. Experimental

In this section, we give a brief introduction to the experimental dataset that we compared to. For more details on the data, readers may refer to the reference [1] for the source of data. Lithium-ion batteries were stored at different temperatures and states of charge (SOC). The capacity of them was recorded around every 30 days to obtain retention rate curves. In our main text, we fit a degradation model (introduced later in Supplemental Section 2) to this experimental degradation data.

1.1. Battery cells

The cells used in the experiment were lithium pouch cells with NMC 622 positive electrode, graphite negative electrode and $LiPF_6$ electrolyte. The capacity of the cell was about 65 Ah. The voltage range limit of the cell was from 3 V (0% SOC) to 4.25 V (100% SOC). Prior to the experiment, the cells were stored for 30 days at 30% SOC to make sure the lithium distribution is uniform in the electrodes.

1.2. Calendar aging

The cells were stored at open circuit conditions for calendar aging tests. As mentioned in the main text, we picked four combinations of SOC and temperature: 10% SOC at 25 °C, 70% SOC at 25 °C, 70% SOC at 45 °C, and 70% SOC at 60 °C. Before storage, the capacity of the cells was measured by the check-up procedure (see Supplemental Section 1.3). In the tests, the storage was interrupted around every 30 days to check the capacity, which was also done by the check-up procedure. After check-up, the cells were set to the specific SOC and stored in a temperature chamber.

1.3. Check-up procedure

The check-up procedure was used to determine C_{std}, the usable capacity of the cells, defined by the discharging the cell at C/3. The cell was first stored at room temperature (25 °C) for 3 hours. Then, it was discharged until the lower cut-off voltage (3 V). Afterwards, it was charged by a constant current (CC) step with C/3 current until the upper cut-off voltage (4.25 V), followed by a constant voltage (CV) step at the voltage until the current drops to C/20. This cycled was repeated three times to check measurement errors in case of outliers. After three cycles, the cell was discharged at C/3 to measure C_{std}. Before the check-up procedure finished, a peak power test was applied for other purposes. The power test is not relevant to our paper and thus will not be discussed here.

To restore SOC conditions after the test, the cell was set to the targeted value $\mathrm{SOC}_{\mathrm{tgt}}$. The cell was charged at C/3 until the amount of charge that flows through the cell reaches $\mathrm{SOC}_{\mathrm{tgt}} \times C_{\mathrm{std}}$.

1.4. Data processing

The capacity of the cells was measured around every 30 days. Due to the limitation of available test channels for check-up and temperature chambers for storage, the time interval between two measurements were not exactly 30 days. For the simplicity of modeling and parameter fitting, we do not consider the variance of intervals and assume that the measurement was conducted exactly every 30 days. We first normalize the raw capacity values by their initial capacity (before storage), and then interpolate the values by cubic curves, as shown in Figure S1. We take data from the curves every 30 days to serve as the experiment data, towards which we fit our degradation model. The data collected from the curves is presented as dots in Figure 3 of the main text.

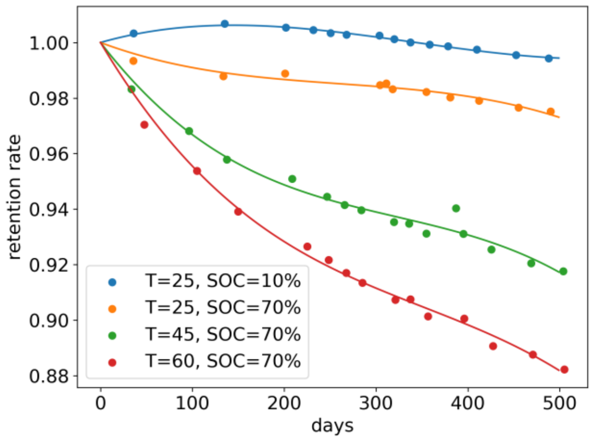

Figure S1. Interpolation of retention rate. The raw data (dots) is interpolated by cubic curves.

2. Degradation model

We use pseudo two-dimensional model [2–4] to simulate a graphite/NMC cell in this study. The degradation mechanisms include solid electrolyte interface (SEI), solvent oxidation and transition metal dissolution.

2.1. Pseudo two-dimensional (P2D) model

Pseudo two-dimensional (P2D) model is a popular model to simulate lithium-ion batteries. There are numerous papers related to this model [2–4]. Here we only give a brief introduction.

The one-dimensional simulation domain is separated into three parts along x axis: negative electrode (graphite), separator, and positive electrode (NMC). In the two electrode domains, lithium concentration in the solid is a function of coordinate x, distance from electrode particle center r, and time t, namely it can be written as $c_{\mathrm{s,k}}(x,r,t)$ where subscript $\mathrm{k} \in \{\mathrm{n,p}\}$ denotes either negative electrode or positive electrode.

The diffusion in the active material particle (graphite or NMC) is given by

$$\frac{\partial c_{\mathrm{s,k}}}{\partial t} = \frac{D_{\mathrm{s,k}}}{r^2}\frac{\partial}{\partial r}\left(r^2 \frac{\partial c_{\mathrm{s,k}}}{\partial r}\right), \quad \text{(S1)}$$

where $D_{\mathrm{s,k}}$ is the lithium diffusivity in the solid. Lithium concentration is related to surface current by

$$-D_{\mathrm{s,k}} \frac{\partial c_{\mathrm{s,k}}}{\partial r}\bigg|_{r=r_{\mathrm{p,k}}} = \frac{i_{\mathrm{Li}}}{F}, \quad \text{(S2)}$$

where $r_{\mathrm{p,k}}$ denotes particle radius, F is Faraday constant, i_{Li} is the current density (per area) of lithium intercalation or deintercalation.

The solid potential, $\Phi_{\mathrm{s,k}}$, is governed by

$$\frac{\partial}{\partial x}\left(\sigma_{\mathrm{s,k}}^{\mathrm{eff}} \frac{\partial \Phi_{\mathrm{s,k}}}{\partial x}\right) = a_{\mathrm{s,k}} i, \quad \text{(S3)}$$

where $\sigma_{s,k}^{eff} = \sigma_{s,k}\varepsilon_{s,k}{}^{1.5}$ is the effective solid conductivity with $\sigma_{s,k}$ being the bulk solid conductivity and $\varepsilon_{s,k}$ being the volume fraction of solid, $a_{s,k} = 3\varepsilon_{s,k}/r_{p,k}$ is the active surface area per unit volume with $r_{p,k}$ being the particle radius, and i denotes the current density (per area) between the solid and the electrolyte, governed by the Bulter-Volmer equation

$$i = i_{0,k}\left[\exp\left(\frac{\alpha_k F\eta}{RT}\right) - \exp\left(\frac{(1-\alpha_k)F\eta}{RT}\right)\right], \tag{S4}$$

where $i_{0,k}$ is the exchange current density, $\alpha_k = 0.5$ is the anodic charge transfer coefficient, R is gas constant, T is temperature, η denotes over-potential. If there is no side reaction, $i_{Li} = i$. Exchange current density $i_{0,k}$ is calculated by

$$i_{0,k} = Fk_{0,k}c_{s,surf,k}^{0.5}c_{e,k}^{0.5}\left(c_{s,max,k} - c_{s,surf,k}\right)^{0.5}, \tag{S5}$$

where $k_{0,k}$ is a reaction coefficient, $c_{e,k}$ denotes lithium concentration in liquid, $c_{s,surf,k}$ denotes concentration at particle surface, $c_{s,max,k}$ denotes the maximum possible lithium concentration.

Over-potential η is calculated by

$$\eta = \Phi_{s,k} - \Phi_{e,k} - U_k^0, \tag{S6}$$

where U_k^0 is the equilibrium potential of active material, $\Phi_{e,k}$ is the electrolyte potential described by

$$\frac{\partial}{\partial x}\left\{-\kappa_{e,k}^{eff}\left[\frac{\partial \Phi_{e,k}}{\partial x} - \frac{2RT}{F}\left(1 + \frac{d\ln f_\pm}{d\ln c_{e,k}}\right)(1-t_+)\frac{\partial \ln c_{e,k}}{\partial x}\right]\right\} = a_{s,k}i, \tag{S7}$$

where $\kappa_{e,k}^{eff} = \kappa_{e,k}\varepsilon_{e,k}{}^{1.5}$ is the effective electrolyte conductivity with $\kappa_{e,k}$ being the bulk electrolyte conductivity, $f_\pm$ is the electrolyte activity coefficient, $c_{e,k}$ is lithium ion concentration in the electrolyte given by

$$\varepsilon_{e,k}\frac{\partial c_{e,k}}{\partial t} + \frac{\partial}{\partial x}\left(-D_{e,,k}^{eff}\frac{\partial c_{e,k}}{\partial x}\right) = \frac{(1-t_+)}{F}a_{s,k}i, \tag{S8}$$

where $D_{e,,k}^{eff} = D_{e,k}\varepsilon_{e,k}{}^{1.5}$ is the effective electrolyte diffusivity with $D_{e,k}$ being the bulk electrolyte diffusivity and $\varepsilon_{e,k}$ being the volume fraction of electrolyte, and t_+ is the lithium-ion transference number.

In the separator domain, lithium is only transferred in liquid, whose governing equations are the same as Eqs. (S7) and (S8) except that i=0.

2.2. Degradation mechanisms

We consider solid electrolyte interface (SEI), solvent oxidation and transition metal dissolution as the degradation mechanisms to account for the capacity decay during storage.

Solid electrolyte interface (SEI). The predominant side reaction occurs in the negative electrode is SEI, where deposit is formed as a product from the reduced electrolyte solvent and consumption of lithium ions and electrons, causing irreversible loss in lithium inventory. Additional current at graphite particle surface is induced [5],

$$i_{SEI} = -e^{-\lambda_{SEI}\delta_{SEI}}Fk_{SEI}c_{EC}\exp\left[-\frac{\alpha_{SEI}F}{RT}\left(\Phi_{s,n} - \Phi_{e,n}\right)\right], \tag{S9}$$

where k_{SEI} denotes the reaction coefficient of SEI formation, c_{EC} is the concentration of ethylene carbonate in the electrolyte. To reflect the decreasing SEI growth rate with increasing SEI thickness (δ_{SEI}) observed in experiments [6], the thickness limiting term of $e^{-\lambda_{SEI}\delta_{SEI}}$ is introduced. The growth of the SEI thickness can be derived from the SEI current using the molar mass and density as shown by [5]

$$\frac{d\delta_{SEI}}{dt} = -\frac{i_{SEI}}{2F}\frac{M_{SEI}}{\rho_{SEI}}, \tag{S10}$$

where M_{SEI} and ρ_{SEI} denote molar mass and density, respectively.

Solvent oxidation. Unlike SEI formation, where solvent reduction occurs at the negative electrode, solvent is oxidized at the positive electrode. Protons (H^+) are produced [5,7], whose reaction rate can be described by

$$i_{\mathrm{oxid}} = \frac{k_{\mathrm{sol}}}{a_{\mathrm{s,p}}} \exp\left[\frac{\alpha_{\mathrm{sol}}F}{RT}\left(\Phi_{\mathrm{s,p}} - \Phi_{\mathrm{e,p}}\right)\right], \tag{S11}$$

where k_{sol} denotes the reaction coefficient of solvent oxidation, and $\alpha_{\mathrm{sol}} = 0.5$.

Transition metal dissolution. Studies have reported acid attack on active material [5]. However, in order to ensure independence among the side reactions, a dissolution mechanism solely dependent on overpotential was used. The rate of change in positive solid phase volume fraction, $\varepsilon_{\mathrm{s,p}}$, is given by [8]

$$\frac{\mathrm{d}\varepsilon_{\mathrm{s,p}}}{\mathrm{d}t} = -\frac{k_{\mathrm{diss}}}{Fc_{\mathrm{s,max,p}}L_{\mathrm{p}}} \exp\left[\frac{\alpha_{\mathrm{diss}}F}{RT}\left(\Phi_{\mathrm{s,p}} - \Phi_{\mathrm{e,p}} - U_{diss}\right)\right], \tag{S12}$$

where k_{diss} denotes the reaction coefficient of transition metal dissolution, $c_{\mathrm{s,max,p}}$ is the maximum lithium concentration of the positive electrode, L_{p} denotes the thickness of positive electrode, $\alpha_{\mathrm{diss}} = 0.5$, and the equilibrium potential is $U_{diss} = 4\ \mathrm{V}$.

Temperature dependence. To account for the temperature dependence of reaction rates, we use Arrhenius relationship to adjust the effective reaction rate, $i_{\mathrm{m}}^{\mathrm{eff}}$, expressed as [7]

$$i_{\mathrm{m}}^{\mathrm{eff}} = i_{\mathrm{m}} \exp\left[\frac{E_{\mathrm{a,m}}}{R}\left(\frac{1}{T_{\mathrm{ref}}} - \frac{1}{T}\right)\right], \tag{S13}$$

where $\mathrm{m} \in \{\mathrm{SEI}, \mathrm{sol}, \mathrm{diss}\}$, i_{m} denotes the current density of side reactions, $E_{\mathrm{a,m}}$ is the activation energy, T is temperature, which is the storage temperature for this study, and T_{ref} is the reference temperature, which is the room temperature at 25°C or 298.15K.

Integration of side reactions. To integrate the three side reactions into the original P2D model described in Supplemental Section 2.1, the induced lithium loss should be added or subtracted from the lithium intercalation or deintercalation [5]. At the negative electrode,

$$i_{\mathrm{Li}} = i - i_{\mathrm{SEI}}. \tag{S14}$$

Meanwhile, the electrolyte volume will be decreased by the growth of SEI layer

$$\varepsilon_{\mathrm{e,n}} = \varepsilon_{\mathrm{e,n}}\big|_{t=0} - \left(\delta_{\mathrm{SEI}} - \delta_{\mathrm{SEI}}|_{t=0}\right)a_{\mathrm{s,n}}. \tag{S15}$$

At the positive electrode

$$i_{\mathrm{Li}} = i - i_{\mathrm{oxid}}. \tag{S16}$$

Transition metal dissolution is reflected in the decreasing solid volume fraction $\varepsilon_{\mathrm{s,p}}$.

2.3. Implementation details

This subsection shows the implementation highlights to build the degradation model to replicate the experiment. The experiment and the model aim to demonstrate the flexibility and powerfulness of the optimization framework instead of finding a perfect model to explain the degradation. Therefore, we made some approximations and simplifications during our implementation.

The equations in the P2D model were solved by the Finite Element Method via COMSOL. For material properties (such as diffusivity, conductivity and maximum lithium concentration), we used the properties of NMC333, graphite and 1 M $LiPF_6$ in 3:7 EC: EMC electrolyte in COMSOL material database. Although these properties may differ from experimental materials, the major degradation mechanisms during calendar aging are the same. Moreover, the optimization algorithm introduced in this paper is versatile to identify side reaction parameters regardless of chosen electrode and electrolyte materials. Due to unspecified values of parameters, several of them were arbitrarily chosen. A list of parameters are shown in Table S1.

Table S1. Major parameters used in the P2D simulation

Symbol	Description	Value	Unit
L_n	Thickness of negative electrode	220	μm
L_p	Thickness of positive electrode	150	μm
L_s	Thickness of separator	30	μm
$\varepsilon_{s,n}$	Solid volume fraction in negative electrode	0.6	
$\varepsilon_{s,p}$	Solid volume fraction in positive electrode	0.5	
$\varepsilon_{e,n}$	Electrolyte volume fraction in negative electrode	0.3	
$\varepsilon_{e,p}$	Electrolyte volume fraction in positive electrode	0.3	
$\varepsilon_{e,s}$	Electrolyte volume fraction in separator	0.45	
$k_{0,n}$	Reaction rate coefficient of negative electrode	3.5×10^{-11}	$m^{2.5}\cdot mol^{-0.5}\cdot s^{-1}$
$k_{0,p}$	Reaction rate coefficient of positive electrode	1×10^{-11}	$m^{2.5}\cdot mol^{-0.5}\cdot s^{-1}$
A_{cell}	Cell cross-sectional area	0.7	m^2
ρ_{SEI}	Density of SEI	1690	$kg\cdot m^{-3}$
M_{SEI}	Molar mass of SEI	0.162	$kg\cdot mol^{-1}$

In the simulation, the steps taken were modified from those taken in the experiment. As mentioned earlier, the peak power test is irrelevant to our paper and is thus removed. Hence, after the 30 min rest at the minimum SOC, the battery cell was charged to its target SOC for calendar aging. The changes in temperature were immediately applied during its step: the gradual increasing and decreasing to the storage and room temperatures were not simulated, because the degradation during this period would be insignificant compared with the long calendar aging.

Supplemental references

1. Rumberg, B., Epding, B., Stradtmann, I., Schleder, M., and Kwade, A. (2020). Holistic calendar aging model parametrization concept for lifetime prediction of graphite/NMC lithium-ion cells. J Energy Storage *30*, 101510.
2. Deng, C., and Lu, W. (2020). Consistent diffusivity measurement between Galvanostatic Intermittent Titration Technique and Electrochemical Impedance Spectroscopy. J Power Sources *473*, 228613.
3. Jokar, A., Rajabloo, B., Désilets, M., and Lacroix, M. (2016). Review of simplified Pseudo-two-Dimensional models of lithium-ion batteries. J Power Sources *327*, 44–55.
4. Wu, B., and Lu, W. (2017). A battery model that fully couples mechanics and electrochemistry at both particle and electrode levels by incorporation of particle interaction. J Power Sources *360*, 360–372.
5. Lin, X., Park, J., Liu, L., Lee, Y., Sastry, A.M., and Lu, W. (2013). A comprehensive capacity fade model and analysis for Li-Ion batteries. J Electrochem Soc *160*, A1701–A1710.
6. Attia, P.M., Chueh, W.C., and Harris, S.J. (2020). Revisiting the $t^{0.5}$ dependence of SEI growth. J Electrochem Soc *167*, 090535.
7. Reniers, J.M., Mulder, G., and Howey, D.A. (2019). Review and performance comparison of mechanical-chemical degradation models for Lithium-ion batteries. J Electrochem Soc *166*, A3189–A3200.
8. Kindermann, F.M., Keil, J., Frank, A., and Jossen, A. (2017). A SEI modeling approach distinguishing between capacity and power fade. J Electrochem Soc *164*, E287–E294.
