# Supplementary material for: A generic battery-cycling optimization framework with learned sampling and early stopping strategies
Source: Patterns (N Y). 2022 Jun 20;3(7):100531. doi: 10.1016/j.patter.2022.100531 (PMC9278511; doi:10.1016/j.patter.2022.100531)
Supplement: Document S2. Article plus supplemental information [file mmc2.pdf]

Patterns

Article

A generic battery-cycling optimization framework with learned sampling and early stopping strategies

Graphical abstract

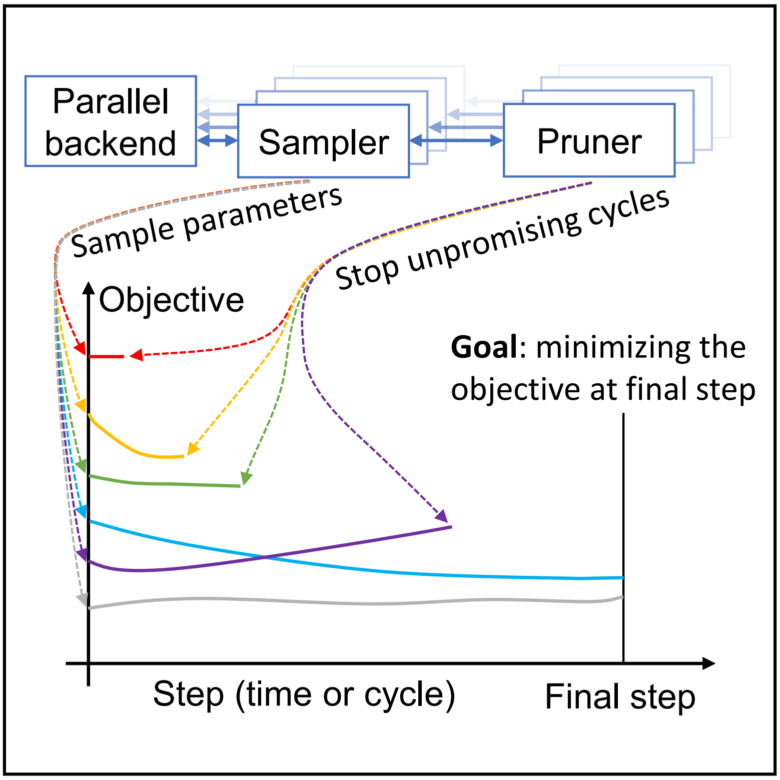

Highlights

- A battery-parameter optimization framework is proposed with a pruner and a sampler
- The framework can optimize categorical, discrete, and continuous variables
- An early-stopping strategy is introduced to reduce the high cycling cost
- Parameter fitting problems are investigated to demonstrate fast optimization

Authors

Changyu Deng, Andrew Kim, Wei Lu

Correspondence

weilu@umich.edu

In brief

Evaluating battery performance typically requires cycling the cells hundreds or thousands of times. Thus, it is time costly to evaluate the effect of battery parameters on its performance and even more expensive to optimize the parameters. We introduced a generic framework leveraging machine learning incorporated with a pruner and a sampler to efficiently optimize the battery parameters. It allows parallel cyclers, stops unpromising cycles, and automatically yields new configurations of parameters. The framework showed excellent results in our demonstration.

Deng et al., 2022, Patterns *3*, 100531
July 8, 2022 © 2022 The Author(s).
https://doi.org/10.1016/j.patter.2022.100531

CellPress

Patterns

CellPress
OPEN ACCESS

Article

A generic battery-cycling optimization framework with learned sampling and early stopping strategies

Changyu Deng,[1] Andrew Kim,[1] and Wei Lu[1,2,3,*]
[1]Department of Mechanical Engineering, University of Michigan, Ann Arbor, MI 48109, USA
[2]Department of Materials Science and Engineering, University of Michigan, Ann Arbor, MI 48109, USA
[3]Lead contact
*Correspondence: weilu@umich.edu
https://doi.org/10.1016/j.patter.2022.100531

THE BIGGER PICTURE There are many parameters to optimize for a battery, in both simulations and experiments, from design to manufacturing. It is time consuming and costly to evaluate the lifetime performance of batteries since it takes a long period to cycle them. We introduce a generic framework leveraging machine-learning algorithms. The framework is designed to optimize battery parameters to enhance cycling performance in a systematic and efficient way, which allows parallel cyclers, stops unpromising cycles, and automatically yields new configurations of parameters. The framework could reduce the average cycling time per battery from years to months/weeks for cycling experiments or from weeks to days/hours for cycling computations. This method is flexible to scale up for many applications, from fundamental research to industrial development in batteries and other similar fields.

1 2 3 4 5 **Development/Pre-production:** Data science output has been rolled out/validated across multiple domains/problems

SUMMARY

Battery optimization is challenging due to the huge cost and time required to evaluate different configurations in experiments or simulations. Optimizing the cycling performance is especially costly since battery cycling is extremely time consuming. Here, we introduce an optimization framework building on recent advances in machine learning, which optimizes battery parameters efficiently to significantly reduce the total cycling time. It consists of a pruner and a sampler. The pruner, using the Asynchronous Successive Halving Algorithm and Hyperband, stops unpromising cycling batteries to save the budget for further exploration. The sampler, using Tree of Parzen Estimators, predicts the next promising configurations based on query history. The framework can deal with categorical, discrete, and continuous parameters and can run in an asynchronously parallel way to allow multiple simultaneous cycling cells. We demonstrated the performance by a parameter-fitting problem for calendar aging. Our framework can foster both simulations and experiments in the battery field.

INTRODUCTION

Energy storage is widely used in many fields, for instance, electrical grid, electric vehicles, portable devices, and so forth. Rechargeable batteries such as lithium ion, lithium oxygen, sodium ion, lead acid, and, more broadly, supercapacitors are highly needed to achieve high capacity and long duration while maintaining low cost.[1,2] An essential task of battery research and development, and for both simulations and experiments, is to optimize the parameters for best performance.

A huge challenge in battery optimization is the high cost to evaluate battery performance. Batteries are expected to have high capacity not only at the beginning but also after thousands of cycles. It is costly and time consuming to wait for battery cycling to collect the data of battery performance after thousands of cycles to compare them and find the optimal battery parameter. What is worse, the space of optimization parameters is often large, and thus a great number of trial queries are necessary to explore different parameter configurations. Imagine we need to optimize 10 battery parameters to maximize the battery

This is an open access article under the CC BY license (http://creativecommons.org/licenses/by/4.0/).

capacity after 1,000 cycles. Evaluating each combination of the parameters may take a few months to cycle the battery, and we need to try 1,024 combinations even if we merely choose two values for each parameter. The total process would require hundreds of years if not using parallel cyclers.

Many works have recently aimed to address such optimization problems. A fast way is to approximate battery behavior by a simple model that is fast to calculate, such as equivalent circuit models[3] and regression models,[4] and then optimize the objective by regular optimization algorithms such as linear programming. However, the accuracy of the results is limited by the simplified models. For example, solid electrolyte interface (SEI) growth has been approximated to be proportional to the square root of time $\sqrt{t}$, but this relation is found to be unjustified in many cases.[5] A more sophisticated method is to use physics-based models, like the pseudo two-dimensional (P2D) model. For example, Lin et al.[6] used gradient descent to minimize capacity fade with power and energy constraints. They simplified the constraints through calculating energy and power by only one-step discharge before degradation, while in real applications, the constraints should be fulfilled for all cycles. Still, they spent 15 days on a partial task. In an experiment, Attia et al.[7] found the optimal charging protocol by Bayesian optimization. They reduced the time from a possible 500 days to 16 days by using a linear regression model[8] to predict battery capacity at the 1,000th cycle based on 100 cycles. Overall, more reliable methods are mandatory to ensure accuracy, especially when dealing with new materials or techniques. However, despite multiple optimization algorithms targeted at expensive objective functions (including the aforementioned gradient descent and Bayesian optimization and other model-based methods such as covariance matrix adaptation evolution strategy[9,10] and self-directed online learning[11]), it becomes a highly costly task to find the optimal parameters even if simplifications and approximations are made due to the complexity of batteries.

To shorten the computational time in simulation or to reduce cost and enable robots[12] in experiments, we require powerful optimization algorithms specifically designed for batteries to address the long-cycling challenge. A special property of battery-cycling optimization is that we evaluate the performance by cycling the battery for a given number of times. Data of the battery behavior, g (such as capacity), is collected during cycling and can be written as a function of cycles, $g(n)$, where n is the number of cycles. The score of the battery is defined on g and can be expressed as $F(g(n))$. For example, F could be the average capacity over the number of cycles or the difference between the model-predicted $g(n)$ and the measured data (parameter fitting). Formally, the single-objective optimization problem can be formulated as

$$\min_{\mathbf{x}} f(\mathbf{x}) = \min_{\mathbf{x}} f(\mathbf{x};\, N) = \min_{\mathbf{x}} F(g(n;\, \mathbf{x})), \qquad \text{(Equation 1)}$$

where $n = 1, 2, \ldots, N$ is the number of steps (steps can be cycles or time), $f(\mathbf{x})$ represents an objective function to be minimized, $\min_{\mathbf{x}} f(\mathbf{x};\, N)$ emphasizes that the minimization is on a parameter vector $\mathbf{x}$ and is related to the total step N, g is a function of n parameterized by $\mathbf{x}$, and F is a functional that calculates the score of the function g. The objective is sometimes to maximize, which can be easily converted to minimize by considering the negative of the original objective. The inequality constraints are

$$IC_i(\mathbf{x}) \le 0,\ i = 1, 2, \ldots, \qquad \text{(Equation 2)}$$

and the equality constraints are

$$EC_j(\mathbf{x}) = 0,\ j = 1, 2, \ldots. \qquad \text{(Equation 3)}$$

The constraints, in some cases, are expressed by functions of cycles like Equation (1).

Current methods directly optimize $f(\mathbf{x}; N)$ without making use of the intermediate information g. Intuitively, one can monitor battery performance during cycling and stop unpromising batteries to make room for new ones. This idea is simple and could save a large amount of time, yet it introduces two fundamental questions: (1) how to determine whether a battery is promising or not. It is challenging even for an expert to make a decision, and now we need a systematic approach to automate decision-making by a computer. (2) How to use the mixed battery data. Some batteries are cycled until the end ($n = N$) so that we know the objective value $f(\mathbf{x};\, N)$, and thus optimization tools can use the values to search for the optimal $\mathbf{x}$. However, many others are early stopped ($n < N$) so that the objective values $f(\mathbf{x};\, N)$ (e.g., the capacity at $N = 1{,}000$ cycles) are unknown for the early-stopped batteries. These incomplete data cannot simply mix with those whose cycling is completed.

In this article, we address the above questions and report a battery-cycling optimization framework covering all of the following highly capable features:

- Able to deal with different types of parameters. Not all parameters are continuous in some cases. Our framework can optimize discrete (e.g., number of cells in a battery pack) and categorical (e.g., electrolyte type) parameters.
- Stop unpromising configurations during cycling. We do not need to cycle all batteries toward the end. Battery data g is measured gradually with cycles. Many batteries, for instance, with low capacity and severe degradation, can be determined to be unpromising during cycling even at the beginning. Thus, the algorithm automatically decides to stop unpromising batteries to make room for new configurations (e.g., new battery parameters) to cycle.
- Asynchronously parallel. Parallel algorithm allows multiple workers (e.g., central processing unit [CPU] cores in simulation or cycling channels in experiment) to cycle batteries at the same time. Asynchrony means each worker will be assigned a new job immediately after a job finishes, with no need to wait for other workers.
- Automatically provide new configurations for cycling based on a machine-learning model trained by all history data. Traditional machine-learning algorithms, such as regression, require parameter set $\mathbf{x}$ and objective $f(\mathbf{x}; N)$ to train a model to learn the input-output relation. If we stop cycling early, intermediate cycling performance $g(n; \mathbf{x})$ cannot be used by traditional machine learning. Our framework enables using all data whether the batteries finish cycling or not.
- Flexible to integrate prior knowledge. For example, we know that the capacity of a battery is a monotonic

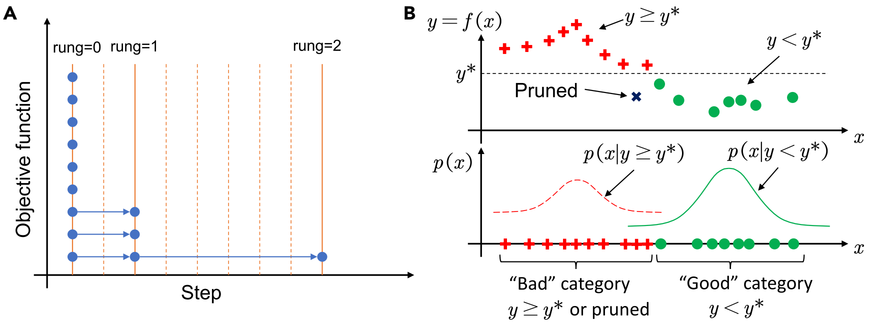

Figure 1. Schematics of the pruner and the sampler

(A) The proposed ASHA promotion scheme. Nine batteries are planned to cycle for up to 9 steps. At each rung, the pruner decides to promote the batteries to the next rung or discard them (stop cycling). The figure shows that only one third of the batteries are promoted at each rung. As a result, only one battery is cycled until the end.

(B) The proposed TPE sampler. In the top plot, the sampler sets a bar y^* and categorizes the historical configurations into a good group (green circles) consisting of points $y_i < y^*$ and bad group (red plus signs) consisting of points $y_i \geq y^*$ or pruned ones. Although the pruned one (blue cross) has a small objective based on intermediate results, it is classified as bad. In the bottom plot, two probability models quantify the probability distribution of the two groups. The x value with high probability in the good group and low probability in the bad group serves as the new query point.

decreasing function (or plus some noise) with respect to cycles, which can be encoded in the algorithm. Other specific knowledge, such as battery-life-prediction models, can be incorporated in the algorithm easily. The algorithm may still work independently without prediction models.

Our framework, which dramatically reduces the optimization time or total cycling time, comprises two major components: a pruner and a sampler.

We use a pruner to determine early stopping of cycling. We adopt Asynchronous Successive Halving Algorithm[13](ASHA), which is a simple and practical hyperparameter tuning method originally proposed for automated machine learning and is suitable for parallelism that exploits early stopping. The basic idea is to only keep a percentage of top candidates. The algorithm decides whether to stop cycling a battery every several cycles, i.e., at each rung (Figure 1A). In our algorithm, ASHA compares the battery with the records of other batteries at the same cycle number. If the performance of this battery is not among the best in all recorded batteries, it will be stopped.

The vanilla ASHA assumes that the best configuration should perform among the top ones after a small number of cycles; otherwise, it would be discarded before cycling until the end. Yet, this is not always the case. A counter example is optimizing average power density with variable discharge current: higher current always induces higher power at the beginning but may cause faster degradation and thus lower power at later stages. To avoid mistakes, decisions cannot be made too early. On the other hand, we want to stop unpromising cycles earlier so we have more budgets for other configurations. Therefore, we incorporated Hyperband[14] with ASHA as a trade-off. Among trials, our approach uses different early-stopping rates, a parameter of ASHA, to control aggressiveness in pruning to cover aggressive pruning strategies and conservative ones (see experimental procedures for details).

We use a sampler to determine new query points based on existing observations. Since the objective function is expensive to evaluate (i.e., needs long cycling), we need to automatically determine the most promising new configurations (i.e., trial battery parameters) for cycling based on historical data. The sampler outputs the configuration **x**, which is most likely to be the best one. As mentioned earlier, the battery data are mixed, i.e., some batteries are cycled to the end (objective values are known) but others are pruned (with only intermediate results). Therefore, instead of a regressor, which correlates configurations with objective functions, we use a classifier, Tree of Parzen Estimators (TPE)[15,16] to model the training data. We choose TPE because (1) it is able to handle categorical and discrete parameters in addition to continuous parameters and (2) it can make use of both finished cycles and pruned cycles. It categorizes observed configurations into a "good" group and a "bad" group by setting a performance threshold, as shown in Figure 1B. Then, it calculates the distribution of two groups and tries to search for configurations with high probability in the good group and low probability in the bad group. Since it only uses ranks instead of absolute-performance values, pruned (early stopped) batteries can be fully utilized by grouping them as bad. Therefore, new query points can be calculated from the historical data including both finished and pruned cycles. Batteries with new configurations are cycled until finished or are stopped by the pruner. Such an iteration continues until the budget is exhausted.

RESULTS

We test the algorithm in parameter-fitting problems for a battery calendar-aging model since we believe parameter fitting is a good candidate to visualize the optimization performance. In other words, the objective function in the optimization problem measures the difference between the measured data and the output of the battery model.

The experimental data, towards which we fit our calendar-aging model, were from lithium-ion pouch cells with graphite and NMC622 electrodes stored at different temperatures and states of charge (SOCs).[17] Four combinations of temperature and SOCs were included: 25°C 10%, 45°C 70%, 60°C 70%, and 25°C 70%. The capacity of fresh cells was measured before aging, and then the remaining capacity was measured every 30 days for a total of 480 days. In total, the experiment obtained 4 fresh-cell-capacity values and 64 degraded-capacity values. The retention rate was obtained from the raw data, as shown in Figure S1. A detailed description of this experimental dataset is presented in the supplemental information.

As for the calendar-aging model, we built a P2D model[2,18] considering three side reactions:[19–21] SEI formation, solvent oxidation, and transition-metal dissolution. The parameters

Table 1. Fitting parameters and results

Parameter	Description	Parameter range	Unit	Case 1 True	Case 1 Estimated	Case 2 Result
k_{SEI}	reaction coefficient of SEI formation	$[10^{-14}, 10^{-12}]$	$m \cdot s^{-1}$	5×10^{-13}	5.18×10^{-13}	1.14×10^{-13}
λ_{SEI}	limiting factor of SEI formation	$[10^5, 10^8]$	m^{-1}	5×10^6	5.24×10^6	3.90×10^6
$E_{a,SEI}$	activation energy of SEI formation	$[10^4, 10^5]$	$J \cdot mol^{-1}$	5×10^4	5.13×10^4	4.36×10^3
k_{sol}	reaction coefficient of SO	[0, 1]	$A \cdot m^{-3}$	0.2	0.23	0
$E_{a,sol}$	activation energy of SO	$[2 \times 10^4, 10^5]$	$J \cdot mol^{-1}$	5×10^4	5.39×10^4	N/A
k_{diss}	reaction coefficient of TMD	0 or $[10^{-9}, 10^{-5}]$	$A \cdot m^{-2}$	10^{-6}	1.07×10^{-6}	0
$E_{a,dis}$	activation energy of TMD	$[5 \times 10^3, 2 \times 10^4]$	$J \cdot mol^{-1}$	8×10^4	8.31×10^4	N/A
SEE	standard error of estimate on capacity	N/A	N/A	N/A	7.95×10^{-4}	5.30×10^{-3}

We consider three side reactions: solid electrolyte interface (SEI) formation, solvent oxidation (SO), and transition-metal dissolution (TMD). SEI will always be included, thus the range of k_{SEI} is positive. In contrast, SO and TMD are optional so their reaction coefficients can be zero (in which case the activation energy is not useful).

and their ranges are shown in Table 1. The details of the degradation model are presented in the supplemental information. Other physical parameters used in the model are fixed, as shown in Table S1.

In our setting, SEI formation will always be included, while the other two reactions are optional and up to the algorithm. Our goal is to find the optimal parameters for the model to match the 64 retention rates (the ratio of degraded capacity to fresh-cell capacity). The objective function penalizes the number of parameters by using the standard error of estimate (SEE):[22]

$$f(\mathbf{x}) = \sqrt{\frac{1}{N-k-1}\sum_{n=1}^{N}[g(n;\mathbf{x}) - g_0(n)]^2}, \quad \text{(Equation 4)}$$

where k is the number of fitting parameters; the capacity of aged cell is normalized by fresh-cell capacity, thus $g(n;\mathbf{x})$ and $g_0(n)$ denote the retention rate from model output and experimental results, respectively; and $N = 64$ is the total number of retention-rate data points measured by the experiment, which is also the number of steps to fit the model. Roughly, SEE is the average difference between the predicted retention rates and the experimental data. Since the retention rate is in the range of [0, 1] and typically close to 1, an SEE value less than 0.01 may be used to indicate that a reasonable fitting is achieved.

To calculate $f(\mathbf{x})$, the degradation at four combinations of temperature and SOCs needs to be computed; each of the combinations needs to calculate the capacity of the fresh-cell- and another 16 degraded-capacity values to fit to experiment data. There is a slight difference between calculating fresh-cell capacity and degraded capacity because the latter also needs to simulate storage, but we ignore this difference when discussing the computation time later since storage simulation takes much less time than simulating capacity measurement. Overall, there are 64 fitting steps or 68 calculation steps. Due to the complexity of the P2D model, each evaluation of $f(\mathbf{x})$ takes about 9 h on our personal computer (CPU: AMD 5950X).

Case 1: Application on simulated data and validation

We first use preset parameters to generate capacity curves from the degradation model and fit the degradation model to the artificially generated data. The preset values (i.e., true parameter values) are presented in Table 1. The fitting error should approach zero when the optimization budget is unlimited. To test whether a feasible solution can be obtained with limited computation, we use a relatively aggressive pruner, ASHA, with minimum step set as 1, namely, a trial battery may be stopped after fitting 1 data point rather than all 64 points. To initialize the algorithm, 20 trials are randomly sampled with 20 sets of parameters. A computation budget of 300 trials is set for each optimization, namely, the algorithm can explore 300 configurations at maximum (although some trials are not complete due to pruning). We use 5 workers and 2 CPU cores per worker to perform asynchronously parallel computations. The optimization is repeated 15 times to get the statistics of the optimization process.

Figure 2 shows the results of the parameter fitting. The simulation data, toward which we fit our model, are plotted as dots in Figure 2A. The optimal fitting curves match perfectly with a small fitting error (SEE = 0.08%). The parameters of the curves, presented in Table 1, are close to our preset values. The query points during this optimization process are plotted in Figure 2B. The size of the parameter space changes because the number of side reactions is automatically selected by the algorithm. Here, we only plot three parameters that appear in all trials, namely, the parameters for SEI formation. These three parameters are normalized by

$$k_{SEI} \leftarrow \log[k_{SEI}/\min(k_{SEI})]/\log[\max(k_{SEI})/\min(k_{SEI})], \quad \text{(Equation 5)}$$

$$\lambda_{SEI} \leftarrow \log[\lambda_{SEI}/\min(\lambda_{SEI})]/\log[\max(\lambda_{SEI})/\min(\lambda_{SEI})], \text{ and} \quad \text{(Equation 6)}$$

$$E_{a,SEI} \leftarrow [E_{a,SEI} - \min(E_{a,SEI})]/[\max(E_{a,SEI}) - \min(E_{a,SEI})], \quad \text{(Equation 7)}$$

so that they are all in the range of [0, 1]. The colors of the spheres or circles reflect the side reactions included in the corresponding trials. The size of the spheres or circles denotes the order of appearance, i.e., large sizes mean that they are sampled at the later stage of optimization. We can see that the sampled points

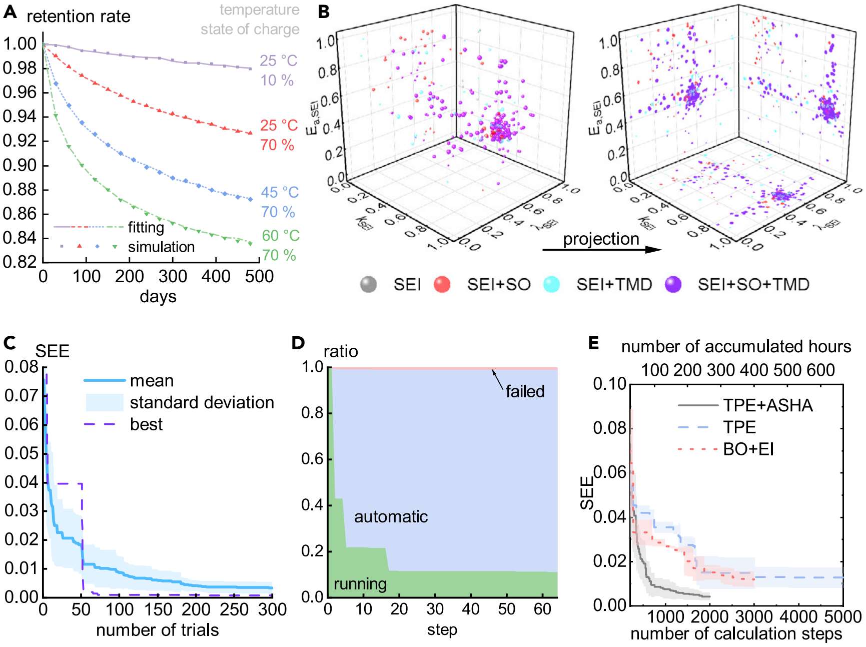

Figure 2. Results of fitting the degradation model to simulation data in case 1

(A) Artificially simulated data (dots) and optimal fitting results (curves).

(B) Scattering of the three parameters during the optimization process whose final degradation curves are presented in (A). The three parameters are rescaled to 0–1 in the plot. The left figure plots the points in a three-dimensional space, and the right figure projects the points onto planes. The spheres and circles are resized according to the order of appearance in optimization, with the smallest one being the first trial and the largest one being the last (300th) trial. The colors represent the side reactions chosen by the algorithm.

(C) SEE (the objective value of optimization) versus given number of trials. Mean and standard deviation are calculated by 15 repeated optimizations, and best denotes the one out of 15 optimizations that produced the optimal parameter, i.e., with minimum SEE after 300 trials, whose degradation curves are presented in (A).

(D) The ratio of trials at three statuses versus the number of fitting steps. Running means the jobs are running, automatic means jobs have been stopped by the algorithm, and failed means jobs have reported errors when solving for degradation curves of the corresponding parameter set. On average, 11% of trials can be calculated until the end, and others are mostly stopped automatically by the algorithm.

(E) SEE (the objective value of optimization) versus number of calculation steps and accumulated hours. Each complete trial consists of 68 calculation steps (64 for fitting data points and 4 calculations to calculate fresh-cell capacity); each calculation step takes 8 min. TPE + ASHA is our proposed method, TPE denotes the method only using the sampler without early stopping, BO + EI indicates Bayesian optimization with expected improvement acquisition function. In BO + EI, the algorithm uses all three side reactions listed in Table 1 instead of automatically selecting side reactions, namely, it solves a simpler problem than the other two. TPE + ASHA was repeated 15 times, and TPE and BO + EI were repeated 3 times. The curves denote the mean, and the shadows denote the standard deviation.

are scattered at first, with various colors representing different choices of side reactions, but then become more concentrated in a small region close to the ground truth, with the violet points meaning that all side reactions are included (which is the same as the ground truth).

Figure 2C shows the statistics of 15 optimizations. The mean SSE is only about 0.3%. In Figure 2D, the average ratios of statuses are presented. Only 11% of trials can be calculated until the end, and the others are mostly stopped automatically by the algorithm. On average, each trial will fit 11.3 data points. In other words, due to early stopping, each optimization is approximately equivalent to 52.9 complete trials. We compare our proposed method (TPE + ASHA) with two baseline methods, as shown in Figure 2E. One method only uses TPE, namely, all trials are calculated until the end without early stopping. We also compare our method with a popular parameter optimization algorithm, Bayesian optimization with expected improvement as the acquisition function (BO + EI). All three algorithms use 5 workers in parallel. BO is implemented via BoTorch.[23] Since it does not support conditional parameters, BO always includes all three side reactions, different from TPE + ASHA and TPE, which choose side reactions automatically. This means that BO solves a simper problem than the other two, which might be the reason that BO performs better than TPE. Our proposed TPE + ASHA method gives the best performance and shows much faster optimization. The accumulated time is estimated by the number of calculation steps times 8 min per step. (Note that the accumulated time is the total amount of time for calculation. The real time is roughly 1/5 of the accumulated time due to parallelization.) If we assume SEE = 0.015 to be the end of optimization, the proposed scheme reduces the computation time from over 12 days (TPE or BO + EI) to 3 days. Time savings will be more prominent with stricter SEE requirements.

Case 2: Application on experiment data

In the previous example, we used artificially generated data to show that the algorithm can find the optimum efficiently. In this example, we demonstrate a real application by leveraging our algorithm to fit the parameters of the degradation model to the experimental data. Different from the previous case, the minimum of the fitting error is unknown. Hyperband is used to allow the algorithm to be less aggressive. Since we expect a reasonably low fitting error, we set a manual rule to prune a trial if the absolute difference between the predicted retention rate and the data is greater than 0.1 at any point. Other settings are the same as the previous case, i.e., 20 initialization trials, 300 maximum trial budget, 5 workers, and 15 repeated optimizations.

Figure 3 shows the results. The experimental data are plotted as dots in Figure 3A. The optimal fitting curves have a good fit with the experiment (SEE = 0.53%). The parameters outputted by the algorithm are shown in Table 1. It can be observed that the algorithm only chooses SEI formation as the side reaction to account for degradation. Figure 3B shows the statistics of 15 optimizations. The mean SSE is only about 0.65%. In Figure 3C, the average ratios of statuses are presented. Only

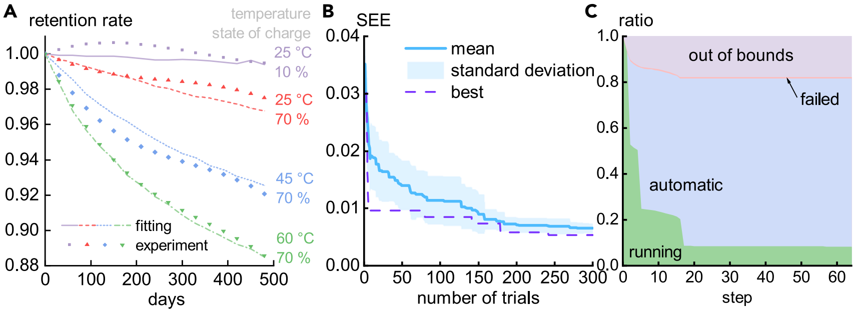

Figure 3. Results of fitting the degradation model to experiment data in case 2

(A) Experiment data (dots) and optimal fitting results (curves).

(B) SEE (the objective value of optimization) versus given number of trials. Mean and standard deviation are calculated by 15 repeated optimizations, and best denotes the one out of 15 optimizations that produced the optimal parameter, i.e., with minimum SEE after 300 trials, whose degradation curves are presented in (A).

(C) The ratio of trials at four statuses versus the number of fitting steps. Running means the jobs are running, automatic means jobs have been stopped by the algorithm, failed means jobs have reported error when solving for degradation curves of the corresponding parameter set, and out of bounds means the difference between the predicted retention rate and the data is greater than 0.1 at any point. On average, 8.1% of trials can be calculated until the end, and the others are mostly stopped automatically by the algorithm.

8.3% of trials are calculated until the end, and the others are mostly stopped automatically by the algorithm. On average, each trial will fit 10.2 data points. In other words, due to early stopping, each optimization process is approximately equivalent to 47.8 complete trials. Compared with case 1, less computation is used, although a more conservative strategy (Hyperband) is implemented, and this may be caused by the nature of the problems. Comparing Figure 2B with Figure 3B, the SEE in the experimental case is flatter than in the simulation case at the later stage since the minimal SEE in experiment is higher than the simulation. As a result, the algorithm is more likely to observe a potentially better solution when fitting to the simulation data and is less likely to be pruned.

DISCUSSION

It is challenging to optimize the parameters of battery cycling in experiments or physics-based simulations. In this article, we introduced a generic framework suitable for battery-cycling optimization. It consists of a pruner and a sampler. We developed the approach of using ASHA (pseudocode in Figure 4) with Hyperband as the pruner and TPE as the sampler. In our demonstration of the calendar-aging parameter-fitting problems, the framework shows excellent performance. It should be noted that while calendar aging is used as an example, the approach can address various complicated cycling problems. This framework can be used for optimization in both simulations and experiments.

There is a trade-off between exploration (conservative pruning) and exploitation (aggressive pruning). Aggressive pruning may be used, for instance, when there is a strong correlation between early and late performance or when we want to obtain a solution quickly without the need of a precise global optimum. On the other hand, if we deal with a problem where initial cell performance reveals little about the final objective performance, a conservative strategy with less or no pruning is necessary. The detailed settings of the framework, such as the hyperparameters, can be adjusted for trade-off between exploration and exploitation based on the problems. For the pruner, we used Hyperband to loop over early-stopping rates to reduce possible pruning mistakes, yet a fixed early-stopping rate will certainly be faster if our domain knowledge gives us confidence in an aggressive strategy. In contrast, if we know that the relation between early-stage performance and final performance is weak, we can force the algorithm to make decisions after more simulation or experimental steps to be conservative. For the sampler, we used TPE, which is versatile and capable to deal with high-dimensional design space, but it may be too aggressive to reach the global optimum. Other sampler algorithms, such as covariance matrix adaptation evolution strategy,[9,10] BO, and reinforcement learning can be attempted as well.

Further work includes embedding battery-specific information into the framework. For example, the current pruner only considers the objective values without trends. Prediction models can be incorporated into the framework.

EXPERIMENTAL PROCEDURES

Resource availability

Lead contact

Further information and requests for resources and reagents should be directed to and will be fulfilled by the lead contact, Wei Lu (weilu@umich.edu).

Materials availability

This study did not generate new unique reagents.

Data and code availability

Optimization algorithms are implemented by Python with Optuna[24] and BoTorch[23] packages. The P2D model was solved by COMSOL Multiphysics automated by MATLAB. All data and code used in this paper are deposited at Zenodo (http://doi.org/10.5281/zenodo.6549835) and GitHub (https://github.com/deng-cy/cycle_opt).

ASHA

ASHA[13] is an extension of Successive Halving Algorithm (SHA).[25,26] Their principles are the same: keep a percentage of top candidates surviving. The pseudocode of ASHA is shown in Figure 4.

Inputs of ASHA are battery parameter vector **x**, maximum number of steps N, and some hyperparameters of the algorithm. Generally speaking, considering the cost of communication between cycler and the computer operating the optimization framework, we run each step n for every one or more cycles. In our two cases, each step corresponds to the prediction of a retention rate, which requires the simulation of a 30-day storage and measurement procedure. The algorithm will not decide early stopping at every step; instead, only certain steps are decisive and called rung. At each rung, a portion (top $1/\eta$) of the batteries are promoted to next rung, i.e., allowed to continue cycling. From our experience, much of the battery information will be revealed at initial stages, thus the intervals between rungs are distributed exponentially, as shown in Figure 1. While the number of steps increases between rungs, the number of cycling batteries decreases, so the total budget roughly remains the same.

Input: configuration $\mathbf{x}$, maximum number of steps N, minimum step r, reduction factor η, minimum early-stoping rate s
Output: total number of finished steps N', objective function $f(\mathbf{x}; N')$

for $n = 1,2, \ldots, N$, **do**
    Calculate $g(n;\mathbf{x})$ // cycle battery to obtain new data
    if *error* // any fatal problem during cycling, for example, the battery is dead or constraints are not met
        return $n-1, f(\mathbf{x}; n-1)$ // return the results of previous step
    end if
    $f(\mathbf{x}; n) \leftarrow F\big(g(n;\mathbf{x})\big)$ //calculate the objective function
    $\mathcal{S}(n) \leftarrow \mathcal{S}(n-1) \cup f(\mathbf{x}; n)$ // store the value in the set
    $rung \leftarrow \max\left\{0, \left\lfloor \log_\eta (n/r) \right\rfloor\right\} - s$ // calculate $rung$ number, $\lfloor\cdot\rfloor$ is the floor function to return greatest integer less than or equal to the input
    if $n = r\eta^{s+rung}$ // if it is this step to decide whether to stop cycling
        $\mathcal{S}_{top}$ ←top_k($\mathcal{S}(n), |\mathcal{S}(n)|/\eta$) // obtain best ($|\mathcal{S}(n)|/\eta$) values from $\mathcal{S}(n)$, $|\mathcal{S}|$ denotes the number of elements in the set $\mathcal{S}$
        if $f(\mathbf{x}; n) \notin \mathcal{S}_{top}$ // not the top values
            return $n, f(\mathbf{x}; n)$
        end if
    end if
end for
return $N, f(\mathbf{x}; N)$

Figure 4. Pseudocode of pruner (ASHA) implemented in this paper

In our demonstration, the algorithm is implemented asynchronously, i.e., a decision will be made based on existing records, even when some configurations are not finished. Therefore, there will be some incorrect promotions to cause actual promotion rates slightly higher than $1/\eta$. We also want to note that it is possible that some previously stopped configurations fall within top ranks later. Then, we can opt to resume cycling these batteries. A drawback of this option is the hassle to store all data, thus such repechage was not implemented in our examples for the sake of memory.

Hyperband

Hyperband[14] tries to resolve the issue about how early we can stop cycling by exploring the early-stopping rate *s* in ASHA. The hyperparameter *s* indicates the average amount of budget we spend per configuration. Intuitively, we want to allocate more resources to distinguish two configurations if they have either high uncertainty or close objective functions. If we have the knowledge about the cycling curves, we can manually choose the optimal *s*. However, the characteristic of curves is up to the task, which makes things challenging and complicated.[27] Unfortunately, in most cases, including our examples, we do not have the available information *a priori*, thus we have to try different early-stopping rates $s = \{s_{max}, s_{max} - 1, \ldots, 0\}$, where $s_{max} = \lfloor \log_\eta N \rfloor$ and $\lfloor \bullet \rfloor$ denotes the floor function that returns the greatest integer less than or equal to the input. It starts from the most aggressive scheme to maximize exploration and ends with all configurations fully cycled.

TPE

The job of a sampler is to point out a new potential optimum according to the query history $\{(\mathbf{x}^{(1)}, y^{(1)}), (\mathbf{x}^{(2)}, y^{(2)}), \ldots, (\mathbf{x}^{(i)}, y^{(i)}), \ldots, (\mathbf{x}^{(m)}, y^{(m)})\}$, where $y^{(i)} = f(\mathbf{x}^{(i)})$. This type of problem is often solved by sequential model-based optimization (SMBO)[28] when the objective function is expensive to evaluate. BO is a subfield of SMBO where the models describe the probability distribution of an objective in unknown space. A strategy in BO is to evaluation points with their EI. For a minimization problem, EI is defined as

$$\mathrm{EI}_{y^*}(\mathbf{x}) = \int_{-\infty}^{y^*} (y^* - y) p(y|\mathbf{x}) dy, \quad \text{(Equation 8)}$$

where y^* is a benchmark value, for instance, the current optimum $\min_j y^{(j)}$. Note that many BO algorithms choose to model $p(y|\mathbf{x})$, yet TPE models $p(\mathbf{x}|y)$ by defining

$$p(\mathbf{x}|y) = \begin{cases} l(\mathbf{x}), & \text{if } y < y^* \\ k(\mathbf{x}), & \text{if } y \geq y^* \end{cases}, \quad \text{(Equation 9)}$$

where y^* is set to be some quantile γ of the observed y values, namely, $p(y < y^*) = \gamma$. $l(\mathbf{x})$ is the density formed by fitting observations $\{\mathbf{x}^{(j)}\}$ whose objective functions are lower than y^* to Gaussian mixture models,[10] and $k(\mathbf{x})$ is the density formed by the rest of the observations.

Using Bayes' rule, Equation (8) becomes

$$\mathrm{EI}_{y^*}(\mathbf{x}) = \int_{-\infty}^{y^*} (y^* - y) \frac{p(\mathbf{x}|y)p(y)}{p(\mathbf{x})} dy. \quad \text{(Equation 10)}$$

Considering

$$p(\mathbf{x}) = \int_{-\infty}^{\infty} p(\mathbf{x}|y)p(y)dy = \gamma l(\mathbf{x}) + (1-\gamma)k(\mathbf{x}) \quad \text{(Equation 11)}$$

and

$$\int_{-\infty}^{y^*} (y^* - y) p(\mathbf{x}|y)p(y)dy = l(\mathbf{x}) \int_{-\infty}^{y^*} (y^* - y)p(y)dy, \quad \text{(Equation 12)}$$

we have

$$\mathrm{EI}_{y^*}(\mathbf{x}) = \frac{l(\mathbf{x}) \int_{-\infty}^{y^*} (y^* - y)p(y)dy}{\gamma l(\mathbf{x}) + (1-\gamma)k(\mathbf{x})} \propto \left[\gamma + \frac{k(\mathbf{x})}{l(\mathbf{x})}(1-\gamma)\right]^{-1}. \quad \text{(Equation 13)}$$

To maximize EI over $\mathbf{x}$, we minimize $k(\mathbf{x})/l(\mathbf{x})$, i.e., choosing points $\mathbf{x}$ with high probability under $l(\mathbf{x})$ and low probability under $k(\mathbf{x})$.

We could see from previous derivations that the relative rank of $y^{(i)}$ from query history matters rather than the absolute value. This property provides us with an advantage to deal with pruned (early stopped) batteries. Note that it is not correct to simply add information of pruned configurations to query history like those completed ones. Pruned batteries, anticipated to perform poorly at the end, may outperform completed ones since the former are only cycled for a small number of times. One way is to ignore all pruned configurations, but this wastes cycling information. Thus, we choose to force pruned configurations within $k(\mathbf{x})$ when categorizing in Equation (9).

SUPPLEMENTAL INFORMATION

Supplemental information can be found online at https://doi.org/10.1016/j.patter.2022.100531.

ACKNOWLEDGMENTS

The authors gratefully acknowledge the support by LG Energy Solution.

AUTHOR CONTRIBUTIONS

Conceptualization, C.D. and W.L.; methodology, C.D.; software, C.D. and A.K.; formal analysis, C.D., visualization, C.D.; writing – original draft, C.D. and A.K.; writing – review & editing, C.D. and W.L.; supervision, W.L.; funding acquisition, W.L.

DECLARATION OF INTERESTS

The authors declare no competing interests.

Received: March 9, 2022
Revised: April 28, 2022
Accepted: May 18, 2022
Published: June 20, 2022

REFERENCES

1. Deng, C., and Lu, W. (2021). A facile process to fabricate phosphorus/carbon xerogel composite as anode for sodium ion batteries. J. Electrochem. Soc. *168*, 080529. https://doi.org/10.1149/1945-7111/ac18e0.
2. Deng, C., and Lu, W. (2020). Consistent diffusivity measurement between galvanostatic intermittent titration technique and electrochemical impedance spectroscopy. J. Power Sources *473*, 228613. https://doi.org/10.1016/j.jpowsour.2020.228613.
3. Bordin, C., Anuta, H.O., Crossland, A., Gutierrez, I.L., Dent, C.J., and Vigo, D. (2017). A linear programming approach for battery degradation analysis and optimization in offgrid power systems with solar energy integration. Renew Energ *101*, 417–430. https://doi.org/10.1016/j.renene.2016.08.066.
4. Maheshwari, A., Paterakis, N.G., Santarelli, M., and Gibescu, M. (2020). Optimizing the operation of energy storage using a non-linear lithium-ion battery degradation model. Appl Energ *261*, 114360. https://doi.org/10.1016/j.apenergy.2019.114360.
5. Attia, P.M., Chueh, W.C., and Harris, S.J. (2020). Revisiting the $t^{0.5}$ dependence of SEI growth. J. Electrochem. Soc. *167*, 090535. https://doi.org/10.1149/1945-7111/ab8ce4.
6. Lin, X., and Lu, W. (2018). A framework for optimization on battery cycle life. J. Electrochem. Soc. *165*, A3380–A3388. https://doi.org/10.1149/2.0741814jes.
7. Attia, P.M., Grover, A., Jin, N., Severson, K.A., Markov, T.M., Liao, Y.-H., Chen, M.H., Cheong, B., Perkins, N., Yang, Z., et al. (2020). Closed-loop optimization of fast-charging protocols for batteries with machine learning. Nature *578*, 397–402. https://doi.org/10.1038/s41586-020-1994-5.
8. Severson, K.A., Attia, P.M., Jin, N., Perkins, N., Jiang, B., Yang, Z., Chen, M.H., Aykol, M., Herring, P.K., Fraggedakis, D., et al. (2019). Data-driven prediction of battery cycle life before capacity degradation. Nat. Energy *4*, 383–391. https://doi.org/10.1038/s41560-019-0356-8.
9. Hansen, N. (2006). Towards a new evolutionary computation. In Advances in the Estimation of Distribution Algorithms, J.A. Lozano, P. Larrañaga, I. Inza, and E. Bengoetxea, eds. (Springer Berlin Heidelberg), pp. 75–102.
10. Hansen, N. (2016). The CMA evolution strategy: a tutorial. Preprint at Arxiv. https://doi.org/10.48550/arxiv.1604.00772.
11. Deng, C., Wang, Y., Qin, C., Fu, Y., and Lu, W. (2022). Self-directed online machine learning for topology optimization. Nat. Commun. *13*, 388. https://doi.org/10.1038/s41467-021-27713-7.
12. Burger, B., Maffettone, P.M., Gusev, V.V., Aitchison, C.M., Bai, Y., Wang, X., Li, X., Alston, B.M., Li, B., Clowes, R., et al. (2020). A mobile robotic chemist. Nature *583*, 237–241. https://doi.org/10.1038/s41586-020-2442-2.
13. Li, L., Jamieson, K., Rostamizadeh, A., Gonina, E., Ben-Tzur, J., Hardt, M., Recht, B., and Talwalkar, A. (2020). A system for massively parallel hyperparameter tuning. Proceedings of Machine Learning and Systems *2*, 230–246. https://doi.org/10.1038/s41586-020-2867-7.
14. Li, L., Jamieson, K., DeSalvo, G., Rostamizadeh, A., and Talwalkar, A. (2017). Hyperband: a novel bandit-based approach to hyperparameter optimization. J. Mach. Learn. Res. *18*, 6765–6816.
15. Bergstra, J.S., Bardenet, R., Bengio, Y., and Kegl, B. (2011). Algorithms for hyper-parameter optimization. In Advances in Neural Information Processing Systems, pp. 2546–2554.
16. Bergstra, J., Yamins, D., and Cox, D. (2013). Making a science of model search: hyperparameter optimization in hundreds of dimensions for vision architectures. In International Conference On Machine Learning, pp. 115–123.
17. Rumberg, B., Epding, B., Stradtmann, I., Schleder, M., and Kwade, A. (2020). Holistic calendar aging model parametrization concept for lifetime prediction of graphite/NMC lithium-ion cells. J. Energy Storage *30*, 101510. https://doi.org/10.1016/j.est.2020.101510.
18. Jokar, A., Rajabloo, B., Désilets, M., and Lacroix, M. (2016). Review of simplified pseudo-two-dimensional models of lithium-ion batteries. J. Power Sources *327*, 44–55. https://doi.org/10.1016/j.jpowsour.2016.07.036.
19. Reniers, J.M., Mulder, G., and Howey, D.A. (2019). Review and performance comparison of mechanical-chemical degradation models for lithium-ion batteries. J. Electrochem. Soc. *166*, A3189–A3200. https://doi.org/10.1149/2.0281914jes.
20. Kindermann, F.M., Keil, J., Frank, A., and Jossen, A. (2017). A SEI modeling approach distinguishing between capacity and power fade. J. Electrochem. Soc. *164*, E287–E294. https://doi.org/10.1149/2.0321712jes.
21. Lin, X., Park, J., Liu, L., Lee, Y., Sastry, A.M., and Lu, W. (2013). A comprehensive capacity fade model and analysis for li-ion batteries. J. Electrochem. Soc. *160*, A1701–A1710. https://doi.org/10.1149/2.040310jes.
22. Smith, G. (2015). Multiple regression. In Essential Statistics, Regression, and Econometrics, Second Edition, G. Smith, ed. (Academic Press), pp. 301–337. https://doi.org/10.1016/b978-0-12-803459-0.00010-8.
23. Balandat, M., Karrer, B., Jiang, D., Daulton, S., Letham, B., Wilson, A.G., and Bakshy, E. (2020). BoTorch: a framework for efficient Monte-Carlo Bayesian optimization. In Advances in neural information processing systems, pp. 21524–21538.
24. Akiba, T., Sano, S., Yanase, T., Ohta, T., and Koyama, M. (2019). Optuna: a next-generation hyperparameter optimization framework. In Proceedings of the 25th ACM SIGKDD international conference on knowledge discovery & data mining, pp. 2623–2631.
25. Karnin, Z., Koren, T., and Somekh, O. (2013). Almost optimal exploration in multi-armed bandits. In Proceedings of the 30th International Conference on Machine Learning Proceedings of Machine, pp. 1238–1246.
26. Jamieson, K., and Talwalkar, A. (2016). Non-stochastic best arm identification and hyperparameter optimization. In Artificial Intelligence and Statistics, pp. 240–248.
27. Ohta, T. (2020). How We Implement Hyperband in Optuna. https://tech.preferred.jp/en/blog/how-we-implement-hyperband-in-optuna/.
28. Hutter, F., Hoos, H.H., and Leyton-Brown, K. (2011). Sequential model-based optimization for general algorithm configuration. In Learning and Intelligent Optimization, C.A.C. Ceollo, ed. (Springer Berlin Heidelberg), pp. 507–523. https://doi.org/10.1007/978-3-642-25566-3_40.

Patterns, Volume *3*

Supplemental information

A generic battery-cycling optimization framework with learned sampling and early stopping strategies

Changyu Deng, Andrew Kim, and Wei Lu

Supplemental Experimental Procedures

Contents

1. Experimental
   1.1. Battery cells
   1.2. Calendar aging
   1.3. Check-up procedure
   1.4. Data processing
2. Degradation model
   2.1. Pseudo two-dimensional (P2D) model
   2.2. Degradation mechanisms
   2.3. Implementation details

1. Experimental

In this section, we give a brief introduction to the experimental dataset that we compared to. For more details on the data, readers may refer to the reference [1] for the source of data. Lithium-ion batteries were stored at different temperatures and states of charge (SOC). The capacity of them was recorded around every 30 days to obtain retention rate curves. In our main text, we fit a degradation model (introduced later in Supplemental Section 2) to this experimental degradation data.

1.1. Battery cells

The cells used in the experiment were lithium pouch cells with NMC 622 positive electrode, graphite negative electrode and $LiPF_6$ electrolyte. The capacity of the cell was about 65 Ah. The voltage range limit of the cell was from 3 V (0% SOC) to 4.25 V (100% SOC). Prior to the experiment, the cells were stored for 30 days at 30% SOC to make sure the lithium distribution is uniform in the electrodes.

1.2. Calendar aging

The cells were stored at open circuit conditions for calendar aging tests. As mentioned in the main text, we picked four combinations of SOC and temperature: 10% SOC at 25 °C, 70% SOC at 25 °C, 70% SOC at 45 °C, and 70% SOC at 60 °C. Before storage, the capacity of the cells was measured by the check-up procedure (see Supplemental Section 1.3). In the tests, the storage was interrupted around every 30 days to check the capacity, which was also done by the check-up procedure. After check-up, the cells were set to the specific SOC and stored in a temperature chamber.

1.3. Check-up procedure

The check-up procedure was used to determine C_{std}, the usable capacity of the cells, defined by the discharging the cell at C/3. The cell was first stored at room temperature (25 °C) for 3 hours. Then, it was discharged until the lower cut-off voltage (3 V). Afterwards, it was charged by a constant current (CC) step with C/3 current until the upper cut-off voltage (4.25 V), followed by a constant voltage (CV) step at the voltage until the current drops to C/20. This cycled was repeated three times to check measurement errors in case of outliers. After three cycles, the cell was discharged at C/3 to measure C_{std}. Before the check-up procedure finished, a peak power test was applied for other purposes. The power test is not relevant to our paper and thus will not be discussed here.

To restore SOC conditions after the test, the cell was set to the targeted value $\mathrm{SOC}_{\mathrm{tgt}}$. The cell was charged at C/3 until the amount of charge that flows through the cell reaches $\mathrm{SOC}_{\mathrm{tgt}} \times C_{\mathrm{std}}$.

1.4. Data processing

The capacity of the cells was measured around every 30 days. Due to the limitation of available test channels for check-up and temperature chambers for storage, the time interval between two measurements were not exactly 30 days. For the simplicity of modeling and parameter fitting, we do not consider the variance of intervals and assume that the measurement was conducted exactly every 30 days. We first normalize the raw capacity values by their initial capacity (before storage), and then interpolate the values by cubic curves, as shown in Figure S1. We take data from the curves every 30 days to serve as the experiment data, towards which we fit our degradation model. The data collected from the curves is presented as dots in Figure 3 of the main text.

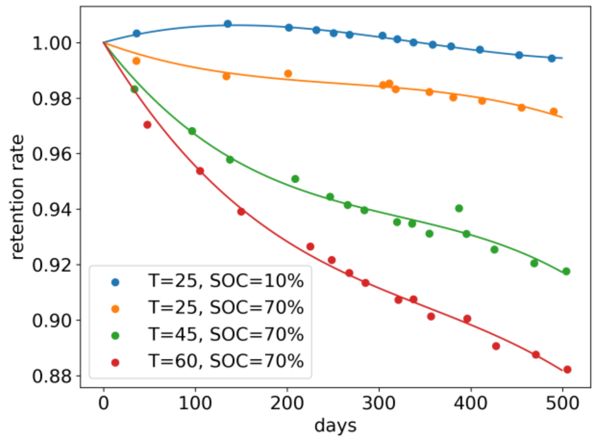

Figure S1. Interpolation of retention rate. The raw data (dots) is interpolated by cubic curves.

2. Degradation model

We use pseudo two-dimensional model [2–4] to simulate a graphite/NMC cell in this study. The degradation mechanisms include solid electrolyte interface (SEI), solvent oxidation and transition metal dissolution.

2.1. Pseudo two-dimensional (P2D) model

Pseudo two-dimensional (P2D) model is a popular model to simulate lithium-ion batteries. There are numerous papers related to this model [2–4]. Here we only give a brief introduction.

The one-dimensional simulation domain is separated into three parts along x axis: negative electrode (graphite), separator, and positive electrode (NMC). In the two electrode domains, lithium concentration in the solid is a function of coordinate x, distance from electrode particle center r, and time t, namely it can be written as $c_{\mathrm{s,k}}(x,r,t)$ where subscript $\mathrm{k} \in \{\mathrm{n,p}\}$ denotes either negative electrode or positive electrode.

The diffusion in the active material particle (graphite or NMC) is given by

$$\frac{\partial c_{\mathrm{s,k}}}{\partial t} = \frac{D_{\mathrm{s,k}}}{r^2}\frac{\partial}{\partial r}\left(r^2\frac{\partial c_{\mathrm{s,k}}}{\partial r}\right), \tag{S1}$$

where $D_{\mathrm{s,k}}$ is the lithium diffusivity in the solid. Lithium concentration is related to surface current by

$$-D_{\mathrm{s,k}}\frac{\partial c_{\mathrm{s,k}}}{\partial r}\bigg|_{r=r_{\mathrm{p,k}}} = \frac{i_{\mathrm{Li}}}{F}, \tag{S2}$$

where $r_{\mathrm{p,k}}$ denotes particle radius, F is Faraday constant, i_{Li} is the current density (per area) of lithium intercalation or deintercalation.

The solid potential, $\Phi_{\mathrm{s,k}}$, is governed by

$$\frac{\partial}{\partial x}\left(\sigma_{\mathrm{s,k}}^{\mathrm{eff}}\frac{\partial \Phi_{\mathrm{s,k}}}{\partial x}\right) = a_{\mathrm{s,k}}i, \tag{S3}$$

where $\sigma_{s,k}^{eff} = \sigma_{s,k}\varepsilon_{s,k}{}^{1.5}$ is the effective solid conductivity with $\sigma_{s,k}$ being the bulk solid conductivity and $\varepsilon_{s,k}$ being the volume fraction of solid, $a_{s,k} = 3\varepsilon_{s,k}/r_{p,k}$ is the active surface area per unit volume with $r_{p,k}$ being the particle radius, and i denotes the current density (per area) between the solid and the electrolyte, governed by the Bulter-Volmer equation

$$i = i_{0,k}\left[\exp\left(\frac{\alpha_k F\eta}{RT}\right)-\exp\left(\frac{(1-\alpha_k)F\eta}{RT}\right)\right], \tag{S4}$$

where $i_{0,k}$ is the exchange current density, $\alpha_k = 0.5$ is the anodic charge transfer coefficient, R is gas constant, T is temperature, η denotes over-potential. If there is no side reaction, $i_{Li} = i$. Exchange current density $i_{0,k}$ is calculated by

$$i_{0,k} = Fk_{0,k}c_{s,surf,k}^{0.5}c_{e,k}^{0.5}\left(c_{s,max,k}-c_{s,surf,k}\right)^{0.5}, \tag{S5}$$

where $k_{0,k}$ is a reaction coefficient, $c_{e,k}$ denotes lithium concentration in liquid, $c_{s,surf,k}$ denotes concentration at particle surface, $c_{s,max,k}$ denotes the maximum possible lithium concentration.

Over-potential η is calculated by

$$\eta = \Phi_{s,k} - \Phi_{e,k} - U_k^0, \tag{S6}$$

where U_k^0 is the equilibrium potential of active material, $\Phi_{e,k}$ is the electrolyte potential described by

$$\frac{\partial}{\partial x}\left\{-\kappa_{e,k}^{eff}\left[\frac{\partial\Phi_{e,k}}{\partial x}-\frac{2RT}{F}\left(1+\frac{d\ln f_\pm}{d\ln c_{e,k}}\right)(1-t_+)\frac{\partial \ln c_{e,k}}{\partial x}\right]\right\} = a_{s,k}i, \tag{S7}$$

where $\kappa_{e,k}^{eff} = \kappa_{e,k}\varepsilon_{e,k}{}^{1.5}$ is the effective electrolyte conductivity with $\kappa_{e,k}$ being the bulk electrolyte conductivity, $f_\pm$ is the electrolyte activity coefficient, $c_{e,k}$ is lithium ion concentration in the electrolyte given by

$$\varepsilon_{e,k}\frac{\partial c_{e,k}}{\partial t} + \frac{\partial}{\partial x}\left(-D_{e,,k}^{eff}\frac{\partial c_{e,k}}{\partial x}\right) = \frac{(1-t_+)}{F}a_{s,k}i, \tag{S8}$$

where $D_{e,,k}^{eff} = D_{e,k}\varepsilon_{e,k}{}^{1.5}$ is the effective electrolyte diffusivity with $D_{e,k}$ being the bulk electrolyte diffusivity and $\varepsilon_{e,k}$ being the volume fraction of electrolyte, and t_+ is the lithium-ion transference number.

In the separator domain, lithium is only transferred in liquid, whose governing equations are the same as Eqs. (S7) and (S8) except that i=0.

2.2. Degradation mechanisms

We consider solid electrolyte interface (SEI), solvent oxidation and transition metal dissolution as the degradation mechanisms to account for the capacity decay during storage.

Solid electrolyte interface (SEI). The predominant side reaction occurs in the negative electrode is SEI, where deposit is formed as a product from the reduced electrolyte solvent and consumption of lithium ions and electrons, causing irreversible loss in lithium inventory. Additional current at graphite particle surface is induced [5],

$$i_{SEI} = -e^{-\lambda_{SEI}\delta_{SEI}}Fk_{SEI}c_{EC}\exp\left[-\frac{\alpha_{SEI}F}{RT}\left(\Phi_{s,n}-\Phi_{e,n}\right)\right], \tag{S9}$$

where k_{SEI} denotes the reaction coefficient of SEI formation, c_{EC} is the concentration of ethylene carbonate in the electrolyte. To reflect the decreasing SEI growth rate with increasing SEI thickness (δ_{SEI}) observed in experiments [6], the thickness limiting term of $e^{-\lambda_{SEI}\delta_{SEI}}$ is introduced. The growth of the SEI thickness can be derived from the SEI current using the molar mass and density as shown by [5]

$$\frac{d\delta_{SEI}}{dt} = -\frac{i_{SEI}}{2F}\frac{M_{SEI}}{\rho_{SEI}}, \tag{S10}$$

where M_{SEI} and ρ_{SEI} denote molar mass and density, respectively.

Solvent oxidation. Unlike SEI formation, where solvent reduction occurs at the negative electrode, solvent is oxidized at the positive electrode. Protons (H^+) are produced [5,7], whose reaction rate can be described by

$$i_{\mathrm{oxid}} = \frac{k_{\mathrm{sol}}}{a_{\mathrm{s,p}}} \exp\left[\frac{\alpha_{\mathrm{sol}} F}{RT}\left(\Phi_{\mathrm{s,p}} - \Phi_{\mathrm{e,p}}\right)\right], \tag{S11}$$

where k_{sol} denotes the reaction coefficient of solvent oxidation, and $\alpha_{\mathrm{sol}} = 0.5$.

Transition metal dissolution. Studies have reported acid attack on active material [5]. However, in order to ensure independence among the side reactions, a dissolution mechanism solely dependent on overpotential was used. The rate of change in positive solid phase volume fraction, $\varepsilon_{\mathrm{s,p}}$, is given by [8]

$$\frac{\mathrm{d}\varepsilon_{\mathrm{s,p}}}{\mathrm{d}t} = -\frac{k_{\mathrm{diss}}}{F c_{\mathrm{s,max,p}} L_{\mathrm{p}}} \exp\left[\frac{\alpha_{\mathrm{diss}} F}{RT}\left(\Phi_{\mathrm{s,p}} - \Phi_{\mathrm{e,p}} - U_{diss}\right)\right], \tag{S12}$$

where k_{diss} denotes the reaction coefficient of transition metal dissolution, $c_{\mathrm{s,max,p}}$ is the maximum lithium concentration of the positive electrode, L_{p} denotes the thickness of positive electrode, $\alpha_{\mathrm{diss}} = 0.5$, and the equilibrium potential is $U_{diss} = 4\ \mathrm{V}$.

Temperature dependence. To account for the temperature dependence of reaction rates, we use Arrhenius relationship to adjust the effective reaction rate, $i_{\mathrm{m}}^{\mathrm{eff}}$, expressed as [7]

$$i_{\mathrm{m}}^{\mathrm{eff}} = i_{\mathrm{m}} \exp\left[\frac{E_{\mathrm{a,m}}}{R}\left(\frac{1}{T_{\mathrm{ref}}} - \frac{1}{T}\right)\right], \tag{S13}$$

where $\mathrm{m} \in \{\mathrm{SEI}, \mathrm{sol}, \mathrm{diss}\}$, i_{m} denotes the current density of side reactions, $E_{\mathrm{a,m}}$ is the activation energy, T is temperature, which is the storage temperature for this study, and T_{ref} is the reference temperature, which is the room temperature at 25°C or 298.15K.

Integration of side reactions. To integrate the three side reactions into the original P2D model described in Supplemental Section 2.1, the induced lithium loss should be added or subtracted from the lithium intercalation or deintercalation [5]. At the negative electrode,

$$i_{\mathrm{Li}} = i - i_{\mathrm{SEI}}. \tag{S14}$$

Meanwhile, the electrolyte volume will be decreased by the growth of SEI layer

$$\varepsilon_{\mathrm{e,n}} = \varepsilon_{\mathrm{e,n}}\big|_{t=0} - \left(\delta_{\mathrm{SEI}} - \delta_{\mathrm{SEI}}|_{t=0}\right) a_{\mathrm{s,n}}. \tag{S15}$$

At the positive electrode

$$i_{\mathrm{Li}} = i - i_{\mathrm{oxid}}. \tag{S16}$$

Transition metal dissolution is reflected in the decreasing solid volume fraction $\varepsilon_{\mathrm{s,p}}$.

2.3. Implementation details

This subsection shows the implementation highlights to build the degradation model to replicate the experiment. The experiment and the model aim to demonstrate the flexibility and powerfulness of the optimization framework instead of finding a perfect model to explain the degradation. Therefore, we made some approximations and simplifications during our implementation.

The equations in the P2D model were solved by the Finite Element Method via COMSOL. For material properties (such as diffusivity, conductivity and maximum lithium concentration), we used the properties of NMC333, graphite and 1 M $LiPF_6$ in 3:7 EC: EMC electrolyte in COMSOL material database. Although these properties may differ from experimental materials, the major degradation mechanisms during calendar aging are the same. Moreover, the optimization algorithm introduced in this paper is versatile to identify side reaction parameters regardless of chosen electrode and electrolyte materials. Due to unspecified values of parameters, several of them were arbitrarily chosen. A list of parameters are shown in Table S1.

Table S1. Major parameters used in the P2D simulation

Symbol	Description	Value	Unit
L_{n}	Thickness of negative electrode	220	μm
L_{p}	Thickness of positive electrode	150	μm
L_{s}	Thickness of separator	30	μm
$\varepsilon_{\mathrm{s,n}}$	Solid volume fraction in negative electrode	0.6	
$\varepsilon_{\mathrm{s,p}}$	Solid volume fraction in positive electrode	0.5	
$\varepsilon_{\mathrm{e,n}}$	Electrolyte volume fraction in negative electrode	0.3	
$\varepsilon_{\mathrm{e,p}}$	Electrolyte volume fraction in positive electrode	0.3	
$\varepsilon_{\mathrm{e,s}}$	Electrolyte volume fraction in separator	0.45	
$k_{0,\mathrm{n}}$	Reaction rate coefficient of negative electrode	3.5×10^{-11}	$m^{2.5}\cdot mol^{-0.5}\cdot s^{-1}$
$k_{0,\mathrm{p}}$	Reaction rate coefficient of positive electrode	1×10^{-11}	$m^{2.5}\cdot mol^{-0.5}\cdot s^{-1}$
A_{cell}	Cell cross-sectional area	0.7	m^2
ρ_{SEI}	Density of SEI	1690	$kg\cdot m^{-3}$
M_{SEI}	Molar mass of SEI	0.162	$kg\cdot mol^{-1}$

In the simulation, the steps taken were modified from those taken in the experiment. As mentioned earlier, the peak power test is irrelevant to our paper and is thus removed. Hence, after the 30 min rest at the minimum SOC, the battery cell was charged to its target SOC for calendar aging. The changes in temperature were immediately applied during its step: the gradual increasing and decreasing to the storage and room temperatures were not simulated, because the degradation during this period would be insignificant compared with the long calendar aging.

Supplemental references

1. Rumberg, B., Epding, B., Stradtmann, I., Schleder, M., and Kwade, A. (2020). Holistic calendar aging model parametrization concept for lifetime prediction of graphite/NMC lithium-ion cells. J Energy Storage *30*, 101510.
2. Deng, C., and Lu, W. (2020). Consistent diffusivity measurement between Galvanostatic Intermittent Titration Technique and Electrochemical Impedance Spectroscopy. J Power Sources *473*, 228613.
3. Jokar, A., Rajabloo, B., Désilets, M., and Lacroix, M. (2016). Review of simplified Pseudo-two-Dimensional models of lithium-ion batteries. J Power Sources *327*, 44–55.
4. Wu, B., and Lu, W. (2017). A battery model that fully couples mechanics and electrochemistry at both particle and electrode levels by incorporation of particle interaction. J Power Sources *360*, 360–372.
5. Lin, X., Park, J., Liu, L., Lee, Y., Sastry, A.M., and Lu, W. (2013). A comprehensive capacity fade model and analysis for Li-Ion batteries. J Electrochem Soc *160*, A1701–A1710.
6. Attia, P.M., Chueh, W.C., and Harris, S.J. (2020). Revisiting the $t^{0.5}$ dependence of SEI growth. J Electrochem Soc *167*, 090535.
7. Reniers, J.M., Mulder, G., and Howey, D.A. (2019). Review and performance comparison of mechanical-chemical degradation models for Lithium-ion batteries. J Electrochem Soc *166*, A3189–A3200.
8. Kindermann, F.M., Keil, J., Frank, A., and Jossen, A. (2017). A SEI modeling approach distinguishing between capacity and power fade. J Electrochem Soc *164*, E287–E294.
